# Ultrasound-Guided Cryoneurolysis for the Treatment of Painful Diabetic Neuropathy of the Foot: A Case Series

**DOI:** 10.7759/cureus.56267

**Published:** 2024-03-16

**Authors:** Igor Filipovski, Rodney A Gabriel, Rene Kestenholz

**Affiliations:** 1 Pain, Copenhagen Cryo Center, Copenhagen, DNK; 2 Anesthesiology, University of California San Diego, San Diego, USA

**Keywords:** nerve block, ultrasound, chronic pain, diabetic neuropathy, cryoneurolysis

## Abstract

Diabetic peripheral neuropathy is one of the most common causes of chronic neuropathic pain. Treatment of peripheral neuropathy has been limited to either treating the underlying cause or using medications, such as tricyclic antidepressants and anticonvulsants, to manage the symptoms. In this case series, we report the use of ultrasound-guided percutaneous cryoneurolysis of the superficial peroneal nerves to treat diabetic neuropathy of the feet. This demonstrates the potential effectiveness and safety of using cryoneurolysis for painful peripheral diabetic neuropathy.

## Introduction

Pain from peripheral neuropathy arises from altered structures of the peripheral nervous system and can be associated with various etiologies [1]. Approximately 15 million people in the United States and Europe have chronic neuropathic pain [2]. Diabetic peripheral neuropathy is one of the most common causes of neuropathic pain. Diabetes impacts about 10% of the population in Europe, and approximately 30% of those patients have diabetic peripheral neuropathy [3,4]. Pain from peripheral neuropathy affects up to 72.3 per 100,000 person-years [5]. Treatment of peripheral neuropathy has been limited to either treating the underlying cause or prescribing medications such as tricyclic antidepressants and anticonvulsants. The use of peripheral nerve injections can diagnose and potentially treat painful peripheral neuropathy; however, when relief is only temporary, options for long-term relief include but are not limited to spinal cord stimulation, dorsal root ganglion stimulation [6], and, potentially, cryoneurolysis. In this case report, we describe the use of cryoneurolysis for the treatment of painful diabetic neuropathy.

## Case presentation

The patients provided written consent for the performance of the procedures and consent for publication, and Institutional Review Board approval was not required. As the patients were treated outside the United States, consent for the Health Insurance Portability and Accountability Act was not obtained. All patients presented in this case series were referred by a general practitioner for painful diabetic neuropathy. The patients had a long-standing history of diabetes and had undergone various treatment modalities for pain without achieving satisfactory outcomes. The individuals referred for pain management underwent thorough evaluation at a diabetes center. Diagnostic evaluations at the diabetes center included comprehensive assessments to confirm the etiology of pain. Following accurate diagnosis establishment and initiation of medication-based interventions to optimize glycemic control, patients were referred for specialized pain treatment.

Case one

A 58-year-old man with a history of diabetes and hypertension presented with chronic pain of the bilateral feet, which had been persisting for seven years despite oral analgesics. Pain intensity using the visual analog scale (VAS) was 80 mm and 60 mm for the right and left feet, respectively. Pain was localized to the dermatome corresponding to the superficial peroneal nerve on both sides. Functional disability before intervention was characterized by persistent foot pain and restricted range of motion, which was indicative of significant impairment. The ineffectiveness of pain control was attributed to the failure of conventional pharmacological interventions, including tricyclic antidepressants, anticonvulsants, and opioids, to alleviate symptoms.

**Figure 1:**
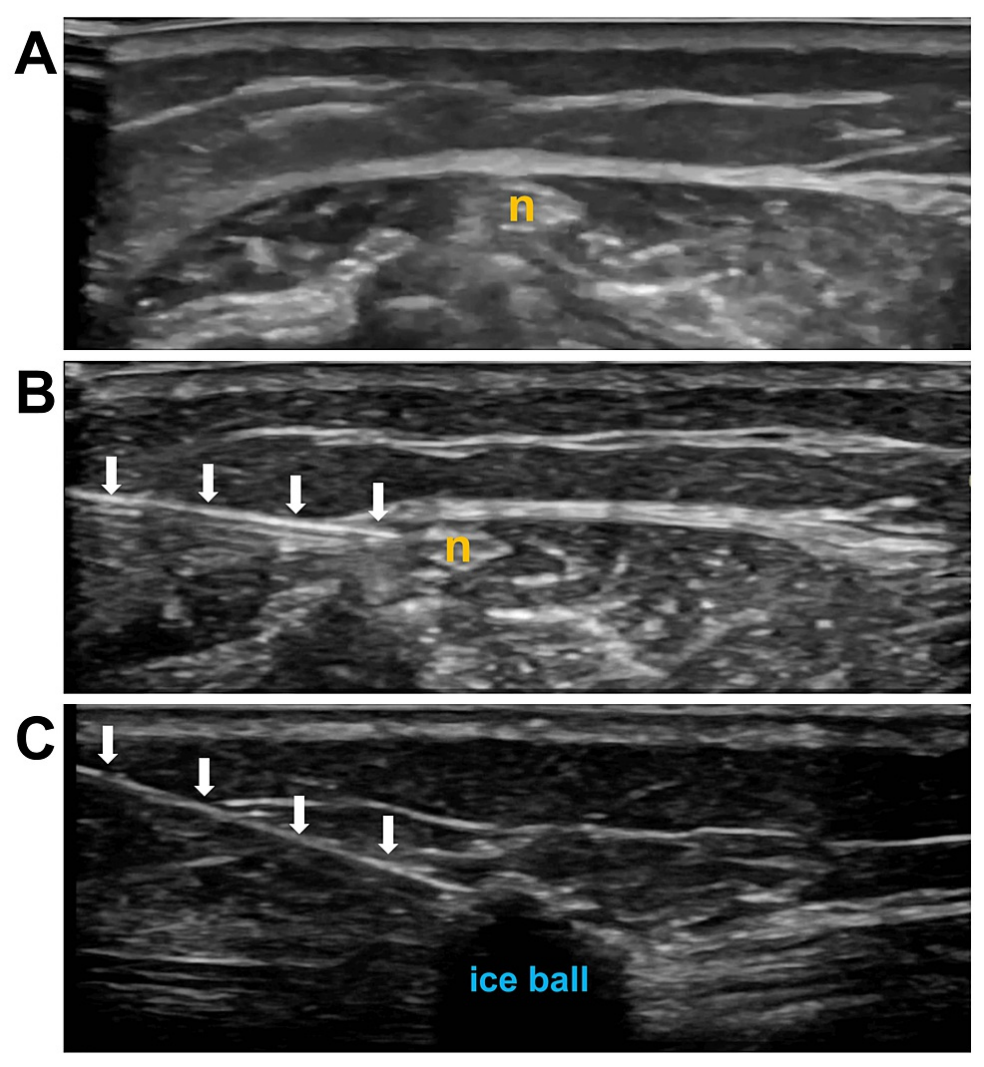
Ultrasound image of cryoneurolysis of the superficial peroneal nerve An ultrasound image of using cryoneurolysis to treat the superficial peroneal nerve for the patient is described in Case 1. A) ultrasound image of the nerve; B) ultrasound image with cryoprobe in view adjacent to nerve; C) ultrasound image of the ice ball covering the superficial peroneal nerve. Abbreviations: n, superficial peroneal nerve; white arrows: needle

The patient underwent an ultrasound-guided diagnostic block of the superficial peroneal nerves bilaterally with lidocaine 1% (1 mL). Within five minutes, the VAS scores decreased from 80 mm to 10 mm for the right foot and from 60 mm to 0 mm for the left foot. The patient was then able to ambulate without pain. At a follow-up appointment, an ultrasound-guided cryoneurolysis treatment was then conducted. This procedure targeted the superficial peroneal nerve on both sides, using a 1.3-mm triangular-shaped needle. The process entailed two cycles of freezing lasting two minutes each, interspersed with a one-minute thawing period (Figure 1).

During the follow-up visit at three weeks, the patient reported experiencing a sudden, shocking pain in his right foot. In response, a right superficial peroneal nerve block was performed with ropivacaine 5 mg/mL solution (1 mL), and the pain resolved. At the six-month follow-up, the patient reported the absence of this recurring pain. Over a subsequent follow-up period spanning five years, the patient remained satisfied with the analgesia, reporting a VAS score of 0 mm and 1-2 mm for the left and right foot, respectively.

Case two

A 70-year-old man with a history of diabetes, hypertension, angina pectoris, and prostate cancer presented with bilateral chronic pain in the feet, which had persisted for eight years and had been refractory to medical management (including tricyclic antidepressants and anticonvulsant medications). Pain was predominantly concentrated on the right foot and exhibited heightened intensity during nighttime hours, which interfered with their sleep pattern. The patient reported VAS scores of 70 mm and 50 mm for the right and left feet, respectively. Pain was localized to the dermatome corresponding to the superficial peroneal nerve bilaterally.

The patient underwent an ultrasound-guided diagnostic block procedure of the superficial peroneal nerve bilaterally with mepivacaine 1% (1 mL). Over a 10-minute period, VAS scores decreased from 70 mm to 10 mm for the right foot and from 50 mm to 10 mm for the left foot. Following this intervention, the patient was able to ambulate without pain while barefoot. Subsequently, at a follow-up appointment, an ultrasound-guided cryoneurolysis treatment of the bilateral superficial peroneal nerves was performed using a round-shaped needle with a diameter of 1.3 mm. The cryoneurolysis process involved subjecting the nerve to two cycles of freezing, each lasting two minutes, interspersed with a one-minute thawing period.

During the follow-up visit at three weeks, the patient reported substantial pain relief, with VAS scores of 30 mm for the right foot and 10 mm for the left foot. The patient was pain-free for three and a half years. However, the pain resurfaced with VAS scores of 60 mm for the right foot and 40 mm for the left foot following this period. In response, an ultrasound-guided cryoneurolysis treatment was repeated five years after the initial treatment, following the same protocol but extending the freezing cycles to three minutes each. During a follow-up visit, the patient reported improved analgesia with VAS scores reduced to 20 mm and 10 mm for the right and left feet, respectively.

Case three

A 54-year-old woman with a history of diabetes, hypertension, and obesity presented with bilateral persistent pain in her feet for four years, which was refractory to analgesic medications. The patient experienced limitations in her ability to walk short distances because of difficulties with balance. She reported pain intensity of VAS scores of 90 mm and 70 mm in her right and left feet, respectively. The pain was primarily localized to the dermatome corresponding to the superficial peroneal nerve on both sides.

During the initial consultation at the clinic, the patient underwent an ultrasound-guided diagnostic block on the bilateral superficial peroneal nerves with lidocaine 1% solution (1 mL). Within five minutes, the VAS scores decreased from 90 mm to 10 mm for the right foot and from 70 mm to 0 mm for the left foot. Following the diagnostic block, this procedure was repeated twice over the course of two weeks.

At a follow-up visit, subsequent treatment involving ultrasound-guided cryoneurolysis was undertaken. This procedure targeted the superficial peroneal nerve on both sides, utilizing a triangular-shaped needle with a diameter of 1.3 mm. The cryoneurolysis process involved subjecting the nerve to two cycles of freezing lasting three minutes each, interspersed with a one-minute thawing period. Notably, the patient experienced immediate improvement in mobility following cryoneurolysis. The sensation normalized in both feet, and the patient reported regaining typical muscle strength and observing normal skin coloration and warmth in her feet.

However, during the follow-up visit three weeks post-treatment, the patient indicated a recurrence of pain in both feet, reminiscent of the initial pain experience. In response, three additional cryoneurolysis sessions, following the same protocol, were conducted over three months. Despite these interventions, the patient reported only transient pain relief. Subsequent follow-up evaluations spanning a period of one year revealed that the patient's pain intensity remained unchanged.

## Discussion

In this case series, we described the use of ultrasound-guided cryoneurolysis of the superficial peroneal nerves to treat chronic pain associated with diabetic neuropathy. In two of the cases, the patients reported significant prolonged analgesia; however, in one of the cases, the benefit was more transient.

Diabetic neuropathy is a late complication of diabetes, and it is a very challenging condition. It includes a broad spectrum of sensory, trophic, and motor disorders of the lower extremities [7]. Traditionally, treatment of peripheral neuropathy has been limited to either treating the underlying cause or using medications such as tricyclic antidepressants, anticonvulsants, and opioids to manage the symptoms [7]. This demonstrates the potential effectiveness and safety of using cryoneurolysis for painful peripheral diabetic neuropathy. However, as a case series of three patients, the study does not necessarily represent diverse experiences.

Cryoneurolysis is a technique of peripheral neurolysis. A probe is placed on the targeted nerve, using ultrasound, and/or a built-in peripheral nerve stimulator. When the probe is in the target location, a gas (carbon dioxide or nitrous oxide) travels down to the center of the probe, where it passes through a tiny opening, which causes the gas to expand and cool the tissues, creating an ice ball. Cryoneurolysis results in a second-degree injury to the peripheral sensory nerve. Wallerian degeneration occurs with exposure to temperatures between -20°C and -88°C, causing a reversible degeneration of the axon beginning at the site of treatment and proceeding distally. Afterward, regeneration of the axon occurs with the Schwann cells undergoing a proliferation and differentiation phase along the endoneurium to reform the scaffolding for the axon. The axons then eventually reinnervate the muscle or sensory receptors. Regeneration occurs at a rate of approximately 1-2 mm per day [8]. Previously, we reported the use of ultrasound-guided cryoneurolysis of the superficial peroneal nerve, sural nerve, and/or neuromas for treating complex regional pain syndrome occurring in the foot and/or ankle [9]. Here, we subsequently report the successful utility of cryoneurolysis of the superficial peroneal nerve for the management of diabetic neuropathy.

## Conclusions

We described the successful treatment of two patients with painful peripheral diabetic neuropathy and a third patient wherein the treatment only had a very short effect for the same condition. We emphasize that the need for interventional techniques (e.g., ultrasound-guided cryoneurolysis) may have important clinical efficacy. This may cut off the process of three major pathophysiological pathways: aberrant inflammatory mechanisms, vasomotor dysfunction, and maladaptive neuroplasticity. Subsequent investigations are imperative to ascertain the efficacy of ultrasound-guided cryoneurolysis as a therapeutic modality for alleviating painful diabetic peripheral neuropathy.
